# Neurosteroids and early-life programming: An updated perspective

**DOI:** 10.1016/j.coemr.2022.100367

**Published:** 2022-08

**Authors:** Ying Sze, Paula J. Brunton

**Affiliations:** Centre for Discovery Brain Sciences, University of Edinburgh, Hugh Robson Building, George Square, Edinburgh, EH8 9XD, Scotland, UK

**Keywords:** Allopregnanolone, Anxiety, GABA receptors, HPA axis, Mass spectrometry, Sex differences, THDOC

## Abstract

Early-life stress can lead to detrimental offspring outcomes, including an increased risk for mood disorders and hypothalamic–pituitary–adrenal axis dysregulation. Neurosteroids bind to ligand-gated neurotransmitter receptors, rapidly modulating neuronal excitability and promoting termination of stress responses. Reduced neurosteroidogenesis underlies some of the aberrant neuroendocrine and behavioural phenotypes observed in adult prenatally stressed rodents. During development, disruptions in neurosteroid generation and action also lead to long-term programming effects on the offspring's brain and behaviour. Here, we review recent advances in the field, focusing on the interaction between neurosteroids and early-life stress outcomes in adulthood and in the perinatal period. We also discuss the direction of future research, with emphasis on quantification methods, sex differences, and neurosteroids as targets for therapeutic intervention.

## Highlights

• Prenatally-stressed offspring display reduced neurosteroidogenesis in the brain.
• Alteration of neurosteroid levels during development has long-term consequences.
• Mass spectrometry methods improve reliability of neurosteroid measurement.
• Neurosteroid regulation of stress responses is sex-, timing- and context-dependent.
• Neurosteroid replacement therapy may be beneficial for stress-related diseases.

## Introduction

Stress experienced during critical developmental periods can result in long-lasting effects that persist into adulthood. This is known as ‘early-life programming’. In humans, early-life stress or adversity is linked to a greater risk for developing metabolic and mood disorders [1]. Similarly, animal models of early-life stress display phenotypes reminiscent of disorders observed in humans, such as anxiety-like behaviour, altered stress-coping behaviour, hypothalamo–pituitary–adrenal (HPA) axis dysregulation and metabolic abnormalities [2,3].

**Figure 1:**
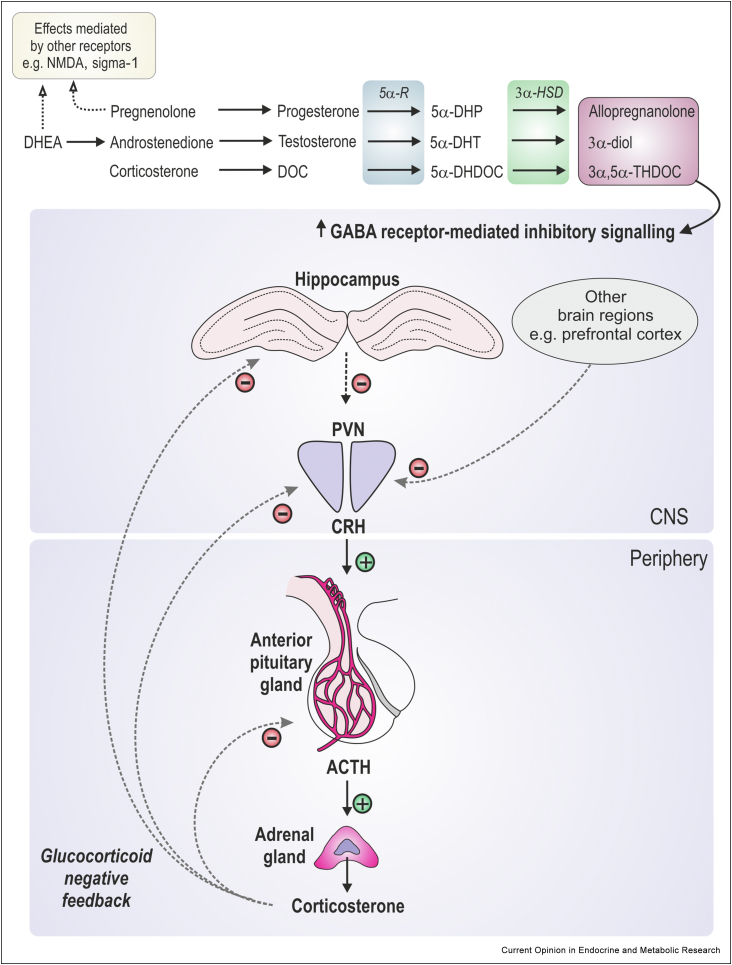
**Neurosteroids and their effects on the HPA axis**. 3α,5α-reduced neurosteroids are converted from their respective precursors via the steroidogenic enzymes 5α-reductase (5α-R) and 3α-hydroxysteroid dehydrogenase (3α-HSD). 3α,5α-reduced steroids are positive allosteric modulators of GABA_A_ receptors and potentiate GABA signaling in brain regions (such as the hippocampus and the prefrontal cortex) that send GABAergic projections to the paraventricular nucleus (PVN) of the hypothalamus. This contributes to the termination of HPA axis stress responses, in addition to glucocorticoid-mediated negative feedback. Other neurosteroids such as pregnenolone and dehydroepiandrosterone (DHEA) can exert fast-acting effects by binding to other ligand-gated ion channels (e.g. NMDA receptors) to alter stress responses or other brain functions, but are beyond the scope of this review. Abbreviations: ACTH, adrenocorticotropin hormone; 3α-diol, 3α-androstanediol; CNS, central nervous system; CRH: corticotropin-releasing hormone; DOC: deoxycorticosterone; 5α-DHDOC, 5α-dihydrodeoxycorticosterone; 5α-DHP, 5α-dihydroprogesterone; 5α-DHT, 5α-dihydrotestosterone; 3α,5α-THDOC, 3α,5α-tetrahydrodeoxycorticosterone.

Neurosteroids are steroids produced locally in the brain that exert modulatory effects on neurophysiological processes, including myelination, neuronal maturation, and neurotransmission. In contrast to steroid hormones which bind to classic intracellular steroid hormone receptors, neurosteroids act directly on ligand-gated ion channels or membrane receptors to exert rapid effects on neuronal signalling [4]. Notably, 3α,5α-reduced neurosteroids, such as allopregnanolone, tetrahydrodeoxycorticosterone (THDOC) and androstanediol (the 3α, 5α-reduced metabolites of progesterone, deoxycorticosterone, and testosterone, respectively; Figure 1), bind allosterically to synaptic and extra-synaptic GABA_A_ receptors, potentiating both phasic and tonic inhibitory signalling, respectively [4, 5, 6, 7]. These neurosteroids have anxiolytic properties, their levels are rapidly increased in the brain following exposure to acute stress, and accumulating evidence indicates they play a key role in aiding in the termination of the HPA axis response by potentiating GABA_A_ receptor-mediated inhibition [4,8, 9, 10, 11](Figure 1). Whereas acute stress increases neurosteroid concentrations, chronic stress typically reduces neurosteroidogenesis [12] and decreased levels of 3α-reduced neurosteroids in the blood and cerebrospinal fluid are reported in individuals with mood disorders [13, 14, 15]. Moreover, treatment with 3α,5α-reduced neurosteroids improves outcomes for a range of stress-related psychiatric disorders, such as anxiety and depression [16], while some antidepressant treatments are associated with increases in central neurosteroid levels [13, 14, 15]. Taken together these findings support a role for neurosteroids in modulating HPA axis stress responses to restore homeostasis, and implicate dysregulated neurosteroid responses in some psychiatric disorders. Although the term ‘neurosteroids’ typically refers to steroids produced *de novo* in the brain [17], steroids generated elsewhere (e.g. adrenal glands, gonads) but transported into the brain to exert neuromodulatory effects (commonly referred to as ‘neuroactive’ steroids) are also considered under the umbrella term ‘neurosteroids’ in this review.

Reduced neurosteroidogenesis, especially allopregnanolone production, has been implicated in the hyperactive HPA axis stress responses and anxious behavioural outcomes of prenatally stressed adult offspring [18,19]. Recent advances in neurosteroid quantification have revealed complexity in their regulation, where alterations in prenatally stressed (PNS) offspring are observed to be region-, sex- and context-dependent [11,20]. Manipulations of neurosteroid action in the pregnant dam or neonate indicate that a successful pregnancy and normal offspring growth and development is contingent on carefully balanced neurosteroid function, where a loss of neurosteroids during pregnancy has negative consequences for the offspring [21, 22, 23∗∗], and reinstating neurosteroid function abrogates these negative offspring outcomes [23,24]. These studies, along with the established role of allopregnanolone in protecting against the effects of stress during pregnancy [25], further reinforce the concept that deficits in neurosteroid production/action following chronic stress during pregnancy can lead to adverse programming outcomes.

## Role of neurosteroids in mediating early life programming effects during adulthood

There is evidence that reduced neurosteroidogenesis contributes to HPA axis dysfunction, anxiety-like behaviour and cognitive deficits observed in adulthood following prenatal stress exposure [18,20,26]. Previous conclusions were largely drawn from reduced gene expression for steroidogenic enzymes (e.g. 5α-reductase) in specific brain regions in PNS animals [18], the finding that deleterious prenatal stress phenotypes can be normalised with exogenous neurosteroid administration [18] or quantification of a limited number of neurosteroids in only one or two gross brain regions [26]. However, recent advances in mass spectrometry have allowed for the direct quantification of multiple neurosteroids in discrete brain regions, revealing finer details regarding their regulation [20].

**Figure 2:**
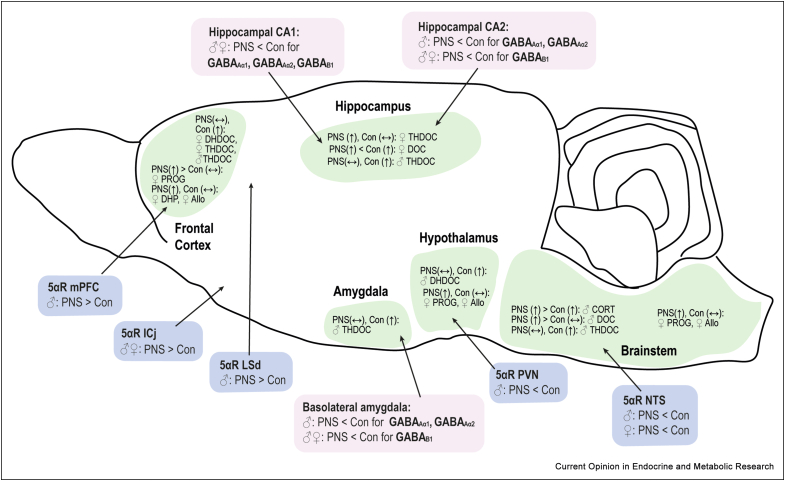
**Changes in central neurosteroid concentrations (in green), 5α-reductase gene expression (in blue) and GABA receptor expression (in pink) in prenatally stressed rat offspring are region- and sex-specific**. For neurosteroid concentrations, arrows indicate greater (↑), or no difference (↔) in neuroactive steroid concentrations following acute swim stress in adulthood compared to basal conditions in control and prenatally stressed male (♂) and female (♀) offspring. In each case, < refers to prenatally stressed (PNS) offspring having lower concentrations/expression levels than control (Con) offspring, and > refers to PNS groups having greater concentrations/expression levels than control offspring. Concentrations of neurosteroids do not differ between PNS and control rats under basal conditions, thus only comparisons between control and PNS groups following acute swim-stress are shown. Abbreviations: Allo, allopregnanolone; Con: control; CORT, corticosterone; DOC, deoxycorticosterone; DHDOC, dihydrodeoxycorticosterone; DHP, dihydroprogesterone; ICj: islands of Calleja in the ventral striatum; LSd: dorsal part of the lateral septum; mPFC, medial prefrontal cortex; NTS, nucleus of the solitary tract; PVN, paraventricular nucleus of the hypothalamus; PNS: prenatally stressed; PROG, progesterone; THDOC, tetrahydrodeoxycorticosterone. Data collated from Brunton et al [18] (5αR mRNA expression); Scott et al [27] (GABA receptor expression) and Sze & Brunton [20] (stress-induced neurosteroid concentrations).

Using a model of maternal psychosocial stress in rats, we found no differences in basal concentrations of neurosteroids in the frontal cortex, hypothalamus, hippocampus, amygdala or brainstem of control and PNS adult offspring [20]. However, when rats experienced an additional acute swim stress during adulthood, which typically increases neurosteroid concentrations in control rats [10,11], deficits in the production of 3α and 3α,5α-reduced metabolites of DOC (DHDOC and THDOC) were detected in all five brain regions investigated in the male PNS offspring, but only in the frontal cortex of the female PNS offspring (Figure 2). These data suggest that differences in neurosteroidogenesis in PNS animals may not only be sex- and region-dependent, consistent with an earlier study in control rats [11], but may also be apparent only in certain contexts (i.e. after an additional stressor during adulthood). These changes, compounded by reduced GABA_A_ receptor expression (targets of 3α,5α-reduced neurosteroids) in the hippocampus and amygdala of PNS offspring [27](Figure 2), likely contribute to the hyperactive HPA axis responses to stress and the anxious phenotype in PNS offspring [28].

In another recent study, where pregnant rats were exposed to metabolic stress in the form of sugar overconsumption [29], changes in central neurosteroid levels in the offspring were also found to be sex- and context-dependent. Under *ad libitum* feeding conditions there was no effect of maternal diet on neurosteroid concentrations in the brain; however, following an additional stressor in adulthood (caloric restriction), brain pregnenolone concentrations were found to be significantly greater in the hippocampus, hypothalamus, medial prefrontal cortex, nucleus accumbens and ventral tegmental area of the adult female, but not male offspring. This may represent a compensatory mechanism, as deficient pregnenolone production in the hypothalamus is reported following a high-fat diet in mice and is associated with impaired cognition [30]. Hence, alterations in neurosteroid synthesis and/or action may contribute to the mechanisms underpinning many outcomes associated with early life stress, including effects beyond those that have been the traditional focus of early life stress research (e.g. mood and cognition). For instance, a role for neurosteroids in modulating the gut–brain axis, which is dysregulated by prenatal stress [31], has recently been explored [30,32].

The effects of prenatal stress on neurosteroid synthesis have also been examined in guinea pigs, which have a steroid profile during pregnancy and parturition more similar to humans than that in rats [33]. Maternal stress using strobe light exposure in late pregnancy results in anxiety-like behaviour and impaired myelination in the juvenile offspring [34, 35, 36]. In this model, 5α-reductase protein expression was not altered in the hippocampus [34]; however, 5α-reductase gene expression was elevated in the cerebellum [37]. Moreover, while brain allopregnanolone concentrations were evidently not affected in juvenile PNS offspring [34], a decrease in circulating allopregnanolone was reported in some studies [36], but not in others [37]. Lower-plasma allopregnanolone concentrations in juvenile offspring are also observed when maternal stress exposure occurs for longer and earlier in pregnancy [35], suggesting that alterations in circulatory allopregnanolone in adulthood is dependent on the timing and duration of the maternal stressor. Interestingly, one study reported that many of the gene expression changes in oligodendrocyte and GABA-related markers (i.e. GABA receptor subunits, GABA synthesis enzymes and cotransporters) in the offspring's brain induced by prenatal stress are absent when prenatal stress is combined with postnatal stress [36], perhaps indicating a predictive adaptive response [38]. Together, these studies indicate a consistent deficit in GABA-mediated processes in PNS guinea pig offspring. However, plasma allopregnanolone measurements tend to be more variable, possibly due to disparities in the age of offspring at the time of sample collection or the measurement methods used (radio-/enzyme-linked immunoassays), and may not accurately reflect changes occurring in the brain.

Alterations in neurosteroid concentrations and the associated deficits in GABAergic signalling in PNS animals make the neurosteroid synthesis pathway an attractive pharmacological target, where programming effects may be reversed by enhancing GABAergic inhibition. Although there has been a lack of new studies in this area, our previous study demonstrated aberrant HPA axis responses in PNS offspring can be normalised by up-regulating expression of the steroidogenic enzymes 5α-reductase and 3α-hydroxysteroid dehydrogenase in the brain or pretreatment with exogenous neurosteroids [18]. Moreover, their therapeutic effect in chronic stress models, where similar phenotypic outcomes are observed as those seen in prenatally stressed animals (e.g. HPA axis dysregulation, depressive-/anxiety-like phenotype), are also apparent [16,39]. However, it should be noted that neurosteroid sensitivity differs according to sex and stress status [18], thus investigation into the downstream response, mediated by GABA_A_ receptors, also warrants further study.

## Role of neurosteroids in programming during the critical development period

During pregnancy, neurosteroids play a protective role where an increase in circulating and central allopregnanolone concentrations dampens maternal HPA axis responses to stress [25]. In the fetus, neurosteroids also protect the developing fetal brain from excessive excitation, and act as morphogens that shape proper development and growth and organisation of neural circuits [40,41]. The effects of neurosteroid loss during this period have been investigated in rodent preterm birth models. Preterm birth is characterised by an abrupt loss of allopregnanolone in the neonatal brain, primarily due to the loss of placental allopregnanolone [42]. This lack of allopregnanolone reduces expression of several GABA_A_ receptor subunits [43] and negatively affects GABA receptor-mediated maturation of oligodendrocytes [44,45]. Accordingly, neurosteroid treatment with ganaxolone, a synthetic allopregnanolone analogue, during the neonatal period reduces the detrimental effects of preterm birth on the offspring [46].

Adverse effects of prenatal stress have also been mimicked in experiments that deplete neurosteroids during critical development periods using finasteride, a 5α-reductase inhibitor. In guinea pigs, maternally administered finasteride results in an anxiety-like phenotype in the female offspring [21], and lower expression of GABA_Aα6_ receptors in the cerebellum [22]. In sheep, concomitant maternal administration of finasteride with gestational stress (isolation and movement restriction) results in greater HPA axis activation in the pregnant ewe [47], which may have deleterious effects on the lamb given the reported adverse effects of excessive glucocorticoid exposure during pregnancy on the fetus [48,49]. In early postnatal life, finasteride administration to rat pups also negatively affects performance in an aversive learning task, but not in a recognition memory task in adulthood [50], further supporting the concept that differences in neurosteroid-mediated regulation of physiology and behaviour are dependent on context (e.g. the presence or absence of stress). While finasteride drastically impacts neurosteroid levels, its use has become contentious due to its side-effects. A study in male rats given sub-chronic finasteride for 20 days reported widespread changes in blood, brain and cerebrospinal fluid concentrations of, not only the neurosteroid metabolites downstream of 5α-reductase, but also in steroid precursors (including those from other steroidogenic pathways), as well as changes in central GABA_A_ receptor expression. Importantly, some of these effects of finasteride persisted even one month after withdrawal [51]. Furthermore, recent studies show finasteride exerts inhibitory effects on the rate-limiting enzyme responsible for adrenaline production, complicating its use in stress-related studies [52]. Given the role of sex steroids in the organisation of the HPA axis during the perinatal period and puberty [53], the use of finasteride during pregnancy or sensitive postnatal developmental windows may lead to sex-specific side-effects; hence, outcomes should be considered with this caveat in mind.

Equally, studies where exogenous neurosteroids are administered during the perinatal period, which generally result in what may be considered positive outcomes, need to be interpreted with caution. This is especially important given endogenous neurosteroid concentrations are dynamic across pregnancy [54,55], and little is known about how exogenous steroids are metabolised locally, transmitted to the fetus, or whether there are other undesired downstream effects. For instance, administration of progesterone to pregnant guinea pigs increases fetal concentrations of circulating allopregnanolone only in the female fetuses, suggesting a sex-specific mechanism in the regulation of steroid transfer and/or metabolism [24]. Maternal allopregnanolone administration also increases fetal allopregnanolone in non-stressed pregnancies, but not in cases of gestational stress exposure [56]. In early post-natal life, allopregnanolone administration is also reported to have wide-ranging effects on adult behaviour, some of which may be considered positive (e.g. lower anxiety-like behaviour), whereas others may be considered harmful (e.g. increased alcohol consumption) [57]. Similarly, whilst ganaxolone rescues some of the adverse effects of preterm birth (mentioned above), side effects such as increased sedation, reduced suckling behaviour and, as a result, increased mortality are also reported [46]. Hence further studies are required to determine the optimal ganaxolone dose that delivers the long-term benefits of neurosteroid replacement, but minimises undesirable side-effects.

Recently, a novel placenta-specific 3α-hydroxysteroid dehydrogenase (3α-HSD; Figure 1) knockout mouse model has been developed, where gene deletion reduces allopregnanolone concentrations in the placenta and fetal brain without affecting the concentration of other neurosteroids, addressing some of the shortcomings of previous studies [23]. Allopregnanolone insufficiency in this model results in sex-specific effects in the offspring, with male offspring from placental 3α-HSD knockout pregnancies displaying increased cerebellar myelination and autism-like behaviours, whereas females show reduced cerebellar myelination, without social behaviour deficits [23]. Allopregnanolone replacement, or administration of the GABA_A_-receptor agonist muscimol, during pregnancy rescues these offspring phenotypes, indicating the effects of allopregnanolone depletion result from a loss in GABA_A_ receptor-mediated allopregnanolone actions in the developing fetus. Importantly, these changes are paralleled in human studies, where premature male, but not female, infants also show increased myelination in the cerebellum [23]. Although these findings are in contrast to guinea pig studies where impaired myelination was reported in preterm male fetuses [24,58], preterm birth also involves a loss of other *in utero* factors which may differ across species [33], again pointing to sex- and context-based regulation of neurosteroid action during critical developmental periods.

Together, these studies where neurosteroid concentrations are manipulated in pregnant dams/fetuses or in early postnatal life, provide further evidence of the critical role neurosteroids play during development and emphasise the potential for external factors (e.g. stress, diet, drugs) that alter neurosteroid production or action at this time to have long-term consequences on the brain and behaviour. Care needs to be taken in interpreting such interventions, taking into account the side-effects of pharmacological inhibition or activation, and the complexities of maternal–fetal steroid transfer and metabolism. Measuring neurosteroids in maternal-fetal-placental triads could go some way to clarifying the full impact of these manipulations [23,24,56]. Recently, levels of endogenous steroids have been reported to differ across different postnatal developmental stages in the mouse cerebellum [59], but as yet no studies have charted the changes in other brain regions, or at earlier developmental stages. Additionally, the downstream mechanisms of how such manipulations affect offspring outcomes in a sex-dependent manner, including the role the placenta might play, requires further study.

## Future directions

### Quantification methods

The ability to accurately quantify concentrations of a panel of different neurosteroids using mass spectrometry [11,20,29] or visualise and map neurosteroids across different brain regions using mass spectrometry-imaging [60] has allowed for thorough investigation of changes in steroids under different conditions and manipulations, and represents an exceptional advance in the field [61]. Nonetheless, there are pitfalls associated with mass spectrometry detection methods as careful tissue preparation, specialised instruments and expertise are required for their proper use, especially in assay validation and quality control [62,63], and it is recognised that not all laboratories have the capabilities for mass spectrometry studies. While immunoassays still have value, it is important that researchers understand their shortcomings in terms of sensitivity and specificity, and recognise that while they can be used to measure steroids of high abundance (e.g. plasma corticosterone concentrations), they may not be the most appropriate method for detecting low levels of neurosteroids in discrete regions of the brain.

Large scale metabolomics, which detect and measure changes in the profiles of steroid metabolites on a systems level, have become an important tool to study pregnancy outcomes in clinical settings [64]. Biomarkers for prenatal disturbances such as intra-uterine growth restriction (IUGR) and gestational diabetes have been identified from a range of different tissues (e.g. maternal blood, urine, amniotic fluid, cord blood) [65]. In women, lower plasma allopregnanolone concentrations in the second trimester of pregnancy are associated with an increased risk of developing clinical post-partum depression [66], and more recently, plasma THDOC has been found to predict gestational age [67]. The role of placental steroid metabolites in predicting pregnancy outcomes is of particular interest [68], especially since the placenta is one of the main sources of steroids during pregnancy, and actively shapes the outcomes of prenatal stress [69].

### Sex differences

Males and females have different risk and resilience profiles to early-life stress, which may change throughout life [70]. Sex differences are largely mediated by gonadal sex steroids, which exert organisational and activational effects via modulation of gene expression patterns through their nuclear receptors, and also through rapid effects on neurotransmission following their metabolism to neurosteroids [71]. During development, sex differences are observed in neurosteroid metabolism and action in the embryonic brain [72]. The placenta also needs to be recognised as sexually dimorphic, and may respond differently to the same environmental insults, leading to different outcomes between the sexes [73]. In adulthood, the downstream actions of neurosteroids are also sex-dependent, with allopregnanolone more effective in suppressing basal hypothalamic and extra-hypothalamic corticotropin-releasing hormone gene expression [74], whereas, in adult prenatally stressed offspring, allopregnanolone is effective in suppressing stress-induced HPA axis activity in female rats, but not males [18]. Overall, the impact of sex on the outcomes of early life stress is complex, and dependent on several factors including the timing and duration of the stressor, as well as the context in which assessments are made [70]. Given that at least some of the outcomes of early-life stress are likely mediated by neurosteroids, sex and age should always be taken into account when administering neurosteroids as an intervention, both in manipulation experiments and in the clinic.

### Neurosteroids as a potential target for intervention

Neurosteroids and their synthetic analogues can restore altered GABAergic neurotransmission and therefore offer potential treatment for mood disorders, including those which may have developmental origins [39]. Modulation of neurosteroid levels during pregnancy and the postpartum period, a time where steroid concentrations undergo dramatic fluctuations, may offer a means through which abrupt changes in neurosteroid function can be buffered in order to reduce the risk of developing mood disorders [75]. Considerable progress has been made since the link between neurosteroids and mood disorders was first identified. Intravenous brexanolone, a synthetic analogue for allopregnanolone, was approved in the USA as the first specific treatment for postpartum depression in 2019 following successful clinical trials [76]. Not only does this represent a significant milestone in the development of neurosteroid-based therapies, but it is especially pertinent in the field of early-life stress, given inadequate maternal care due to postpartum depression can also illicit programming effects on the offspring [77]. More ‘neurosteroid-replacement therapy’ drugs are entering the pipeline – ganaxolone was recently approved in the USA for the treatment of seizures [78] and clinical trials for an orally-active allopregnanolone formulation, SAGE-217, for the treatment of major depressive disorder are currently under way [79]. Various alternatives other than the steroid ligand itself are also being explored as possible targets, such as translocator protein (TSPO) – which facilitates cholesterol transport across the mitochondrial membrane and stimulates neurosteroidogenesis and the endocannobinoid system – a recently realised target for promoting neurosteroidogenesis [80]. Concurrent with developing new therapies, more awareness on how metabolism to neurosteroids may influence the effects of current steroid therapies is needed. For instance, progesterone supplementation is administered to pregnant women to reduce the risk of preterm birth [81], but how such a treatment may indirectly affect maternal mood or prenatally program the fetus via metabolism to other neuroactive steroids (e.g. allopregnanolone), has been largely overlooked and needs revisited.

## Conclusions

**Figure 3:**
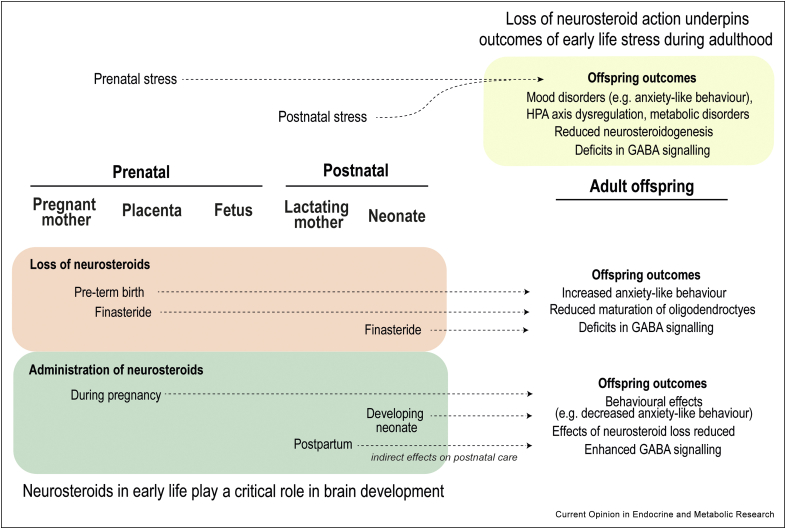
**Overview of the role for neurosteroids in early life and how they impact offspring outcomes**. In prenatally stressed offspring, reduced neurosteroidogenesis underlies some of the outcomes associated with early life stress. Manipulation of neurosteroids during the perinatal period also has effects on the offspring, with the loss of neurosteroids generally resulting in what may be considered less favourable outcomes, and administration of neurosteroids following perturbations ameliorating adverse effects. In most cases, altered GABA signalling is also found as a downstream effect of early life stress exposure/neurosteroid manipulations, in which 3α,5α-neurosteroids have the potential to modulate.

Collectively, the evidence indicates that dysregulation of neurosteroid pathways are involved in mediating the outcomes of early-life stress, both during the critical perinatal period and in adulthood (Figure 3). There is promise for neurosteroids/neurosteroid analogues and their effectors as potential targets for intervention, but more awareness of how sex-, region- and stress-context differences can alter the extent of their influence is needed. Advances in mass spectrometric detection methods has allowed for direct measurements of neurosteroid concentrations in discrete brain regions, though the available information tends to be from adult offspring, with far less known about steroid dynamics across the perinatal period. Further studies investigating the effects of neurosteroid manipulation on the maternal-placental-fetal triad are needed to better understand their role in fetal programming.

## Conflict of interest statement

Nothing declared.
